# Revisiting the role of mesenchymal stem cells in tuberculosis and other infectious diseases

**DOI:** 10.1038/s41423-023-01028-7

**Published:** 2023-05-12

**Authors:** Annu Devi, Isha Pahuja, Shashi Prakash Singh, Akanksha Verma, Debapriya Bhattacharya, Ashima Bhaskar, Ved Prakash Dwivedi, Gobardhan Das

**Affiliations:** 1 Special Centre for Molecular Medicine, Jawaharlal Nehru University, New Delhi, India; 2 Immunobiology Group, International Centre for Genetic Engineering and Biotechnology, New Delhi, India; 3 Department of Molecular Medicine, Jamia Hamdard University, New Delhi, India; 4 Centre for Biotechnology, Siksha O Anusandhan University, Bhubneshwar, Odisha India

**Keywords:** Mesenchymal Stem Cells (MSCs), Macrophages, Tuberculosis, Infectious Diseases, T cells, Immunomodulation, Immunotherapy., Tuberculosis, Infection

## Abstract

Mesenchymal stem cells (MSCs) play diverse roles ranging from regeneration and wound healing to immune signaling. Recent investigations have indicated the crucial role of these multipotent stem cells in regulating various aspects of the immune system. MSCs express unique signaling molecules and secrete various soluble factors that play critical roles in modulating and shaping immune responses, and in some other cases, MSCs can also exert direct antimicrobial effects, thereby helping with the eradication of invading organisms. Recently, it has been demonstrated that MSCs are recruited at the periphery of the granuloma containing *Mycobacterium tuberculosis* and exert “Janus”-like functions by harboring pathogens and mediating host protective immune responses. This leads to the establishment of a dynamic balance between the host and the pathogen. MSCs function through various immunomodulatory factors such as nitric oxide (NO), IDO, and immunosuppressive cytokines. Recently, our group has shown that *M.tb* uses MSCs as a niche to evade host protective immune surveillance mechanisms and establish dormancy. MSCs also express a large number of ABC efflux pumps; therefore, dormant *M.tb* residing in MSCs are exposed to a suboptimal dose of drugs. Therefore, it is highly likely that drug resistance is coupled with dormancy and originates within MSCs. In this review, we discussed various immunomodulatory properties of MSCs, their interactions with important immune cells, and soluble factors. We also discussed the possible roles of MSCs in the outcome of multiple infections and in shaping the immune system, which may provide insight into therapeutic approaches using these cells in different infection models.

## Introduction

Mesenchymal stem cells (MSCs) are nonhematopoietic adult stem cells that possess self-renewal and multidifferentiation potential. These cells are involved in various physiological processes, such as tissue homing, regeneration, and immunomodulation. In 1867, Cohnheim speculated on the presence of MSCs in the bone marrow for the first time. He observed the presence of nonhematopoietic stem cells in the bone marrow that could be differentiated into fibroblasts during tissue repair [1]. These cells were later identified by Freidenstein, a pioneer in the field, in 1970 when he demonstrated the presence of a nonhematopoietic bone marrow cell population in cell cultures. He observed a heterogeneous population of adherent cells with fibroblastic morphology that developed into clonogenic colonies termed colony-forming unit-fibroblasts (CFU-Fs) [2]. The term MSCs was coined by Caplan in 1991 [3].

**Fig. 1:**
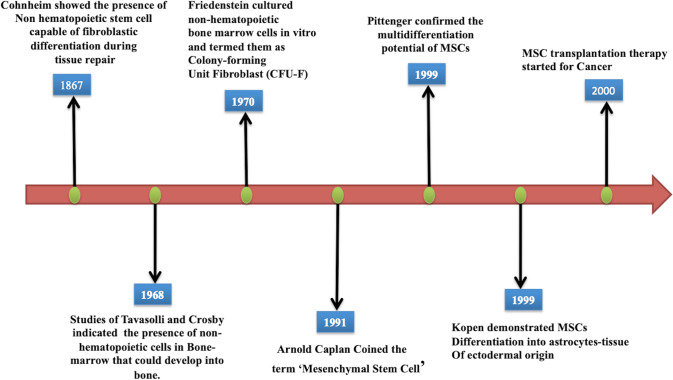
Timeline of the characterization of MSCs

Later, Pittinger et al. demonstrated the multidifferentiation potential of MSCs into adipocytes, chondrocytes, and osteocytes [4]. Furthermore, the ability of MSCs to differentiate into cells other than the mesodermal lineages was verified when these cells were injected into the lateral ventricle of neonatal mice and differentiated into astrocytes [5] (Fig. 1).

There have been many debates on using the term mesenchymal stem cells for these cell populations. Many have suggested the use of alternate terms, such as multipotent stromal cells or mesenchymal stromal cells. In 2005, the International Society for Cellular Therapy (ISCT) defined multipotent mesenchymal stromal cells as cell populations isolated from any tissue that has fibroblast-like-plastic adherent properties. In contrast, mesenchymal stem cells are cells that possess stem cell properties [6]. In 2016, the ISCT established minimum criteria for defining mesenchymal stromal cells. (i) The cells must be plastic-adherent when cultured in standard conditions. (ii) The cells must express the surface molecules CD73, CD90, and CD105 and lack expression of CD34, CD45, CD14, CD19, and HLA-DR. (iii) The cells must differentiate into adipocytes, osteoblasts, and chondroblasts in vitro [7].

Interestingly, in 2010, Caplan suggested renaming these cells ‘Medicinal signaling cells.’ He suggested that these cells were present not only in mesodermal tissues but also in every vascularized tissue. In addition, MSCs have strong therapeutic potential because they can home to tissues and secrete numerous immunomodulatory and signaling molecules [8].

MSCs are mostly isolated from the bone marrow and represent a very low population of approximately 1 in 10,000 nucleated cells. Apart from the bone marrow, these cells have been successfully isolated from different tissues, such as adipose tissue, amniotic fluid, dental pulp, the placenta, the endometrium, umbilical cord, umbilical cord blood, Wharton jelly, and peripheral blood [9, 10].

The surface expression of different receptors is an important criterion for characterizing MSCs. Human MSCs express surface molecules such as CD44, CD71, CD73, CD90, and CD105. These cells are also positive for adhesion molecules such as CD106, CD166, ICAM-1, and CD29. All human MSCs are negative for hematopoietic lineage markers such as CD34, CD45, CD14, and CD11, as well as costimulatory molecules such as CD80, CD86, and CD40 [2]. In addition, human MSCs are said to be negative for MHC-II and show intermediate expression of MHC-I. The presence and expression patterns of these receptors on MSCs may vary depending on the tissue of origin and the species from which they are isolated. The MSC population isolated from tissues is highly heterogeneous, and cells have different lineage commitments defined by their in vivo environment. However, few of these cells are stem cells that can be characterized by specific markers known as stemness markers, which include stro-1, SSEA-4, CD106, and CD146 [9].

**Fig. 2:**
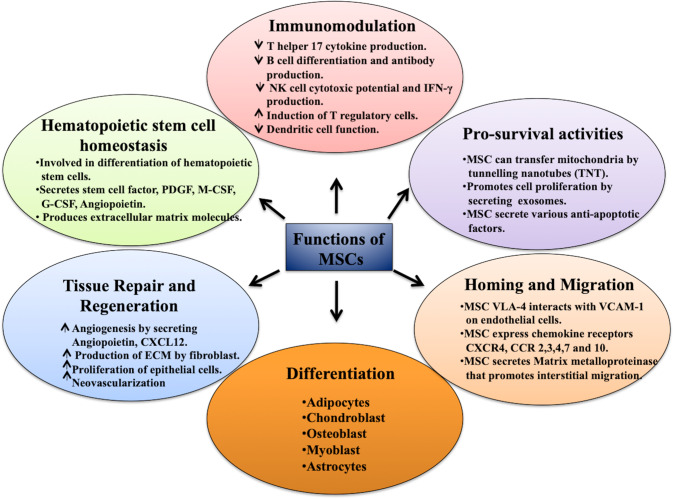
Schematic representation of the diverse functions of MSCs

The different crucial functions of MSCs have led to their use in various studies of degenerative diseases, tissue engineering, and immune disorders, and multiple preclinical and clinical trials are underway [11]. MSCs can differentiate into multiple cell types and are used for various tissue regeneration and tissue engineering studies. Much effort is being spent on culturing MSCs in 3D cell culture models to differentiate these cells into different tissues of the skeletal system and use them to treat various deformities or other related complications. MSCs play a significant role in maintaining the homeostasis and differentiation potential of the hematopoietic stem cell (HSC) pool in the bone marrow. MSCs secrete various growth factors and chemokines, such as platelet-derived growth factor (PDGF), stem cell factor, macrophage colony-stimulating factor (M-CSF), granulocyte-CSF (G-CSF), erythropoietin, CXCL12, and CCL5. Many of these factors are critical for differentiating HSCs into various lineages. CXCL12 produced by MSCs is crucial for maintaining HSCs [12]. Some factors are important for inhibiting apoptosis and stimulating proliferation and angiogenesis [13, 14]. MSCs are essential in wound healing and regeneration, contributing to multiple aspects of this process. MSCs facilitate the process of wound healing through multiple anti-inflammatory effects, such as the polarization of macrophages to the M2 phenotype and the production of various anti-inflammatory cytokines. In addition, MSCs and their secreted exosomes promote the production of extracellular matrix by fibroblasts and the proliferation of epithelial cells. MSCs also promote neovascularization by secreting many factors that contribute to angiogenesis, such as CXCL12, VEGF, and EGF [15]. The homing of MSCs to injured sites is critical for processes such as wound healing and is also used for various stem cell-based therapies for regeneration. Similar to leukocytes, MSCs have the ability to migrate to tissues in the case of injury and inflammation in response to chemokine gradients. The expression of various chemokine receptors, such as CXCR4 and CCR2, −3, −4, −7, and −10, by MSCs may play a crucial role in this process. In the blood circulation, MSCs adhere to the endothelial lining of blood vessels through the interactions of VLA-4 molecules expressed on the surface of MSCs with VCAM-1 on endothelial cells. This is followed by transendothelial and interstitial migration to the injured sites. MSCs secrete various matrix metalloproteinases, such as MMP-1, −2, −3, and −9, which help in interstitial migration [13, 16, 17]. The exact mechanisms and molecules involved in MSC homing are still being investigated, since it is critical to use MSCs for various therapies. MSCs also play a critical role in modulating cell survival through processes such as apoptosis. MSCs have been reported to prevent cell death by multiple mechanisms. MSCs can secrete various cytokines, such as IL-6 and IL-10, that have been reported to directly affect cell death pathways. In addition to cytokines, MSCs can secrete extracellular vesicles containing biomolecules such as proteins and miRNAs that can modulate various molecules involved in cell death. MSCs can prevent cell death by expressing connexin (Cx)-43 gap junctions, which may occur through the exchange of calcium ions. It has also been reported that MSCs with higher levels of Cx-43 junctions have higher expression of anti-apoptotic molecules such as Bcl-2, thus facilitating cell survival. MSCs also enhance cell survival through the transfer of mitochondria via channels known as tunneling nanotubes (TNTs) [18, 19]. The immunomodulatory properties of MSCs have attracted much attention in the scientific community. Numerous studies have demonstrated that MSCs can interact with and modulate the functions of different immune cells, such as T cells, B cells, macrophages, dendritic cells, and NK cells [20–22] (Fig. 2).

In this review, we discussed the functional role of MSCs in TB. MSCs play a significant role in *M.tb* dormancy. We have examined the crucial interactions and cellular adaptations of *M.tb* after they infect MSCs. Moreover, the potential of MSCs in therapeutic and diagnostic interventions has been evaluated. Apart from *M.tb*, we have also examined the interplay of MSCs with other bacterial, viral, and protozoan diseases. Since multiple immune cells are involved during the development of TB and diverse interactions among them are critical in defining the outcomes of the infection, we have also discussed the mechanisms by which MSCs interact with and modulate the different types of immune cells in the body.

## The role of MSCs in tuberculosis

*Mycobacterium tuberculosis (M.tb)*, the etiological agent of tuberculosis (TB), is the second leading cause of global mortality by any infectious pathogen after the recent COVID-19 pandemic. According to WHO reports, approximately 10.6 million cases of TB were reported in 2021. A total of 1.6 million deaths due to TB occurred, of which 1.4 million were among HIV-negative individuals and 187,000 were among HIV-positive people. The number of deaths and drug-resistant cases have increased since 2019 [23]. Approximately one-quarter of the world’s population is infected with TB. In most TB cases, the bacteria remain latent without any symptoms. These latent individuals pose a significant challenge to eradicating TB, since dormant *M.tb* can reactivate under immunosuppressive conditions such as AIDS and malnutrition.

*M.tb* primarily infects the lungs, which is known as pulmonary TB, although extrapulmonary TB is seen in other organs, such as bone, kidney, and brain. The infection typically occurs through the inhalation of aerosols containing bacteria. The bacteria then reach the alveoli in the lungs, where alveolar macrophages phagocytose the pathogen through receptor-mediated endocytosis [24, 25]. Other antigen-presenting cells, such as dendritic cells, phagocytose *M.tb* and present them on MHC-II, leading to the development of CD4^+^ T-cell responses.

The major CD4^+^ T helper 1 (Th1) subset is involved in protecting against *M.tb*, and these cells secrete the major cytokine IFN-γ [26]. IFN-γ activates macrophages, thereby enhancing their antimycobacterial mechanisms, such as increasing phagolysosomal killing and inducing the production of reactive oxygen species (ROS) and reactive nitrogen species (RNS), which can ultimately lead to the killing of *M.tb* [27].

During evolution, *M.tb* has acquired several strategies to evade host immune responses and establish a persistent infection. These strategies include inhibiting phagolysosomal fusion and the acidification of lysosomes, inhibiting antigen presentation and autophagy, or cytosolic escape [28]. Major studies of *M.tb* have focused on its interaction with macrophages. The interactions of many other cell types, such as alveolar epithelial cells and human lymphatic endothelial cells (hLECs), with *M.tb* have been studied. Recently, our group and others have demonstrated that *M.tb* uses MSCs as a reservoir during latency [29].

### MSCs as reservoirs for dormant *M.tb*

Dormancy is a crucial mechanism for immune evasion and protection from antitubercular drugs, leading to the establishment of latent infection. During dormancy, *M.tb* exhibits low metabolic activity, does not replicate, and is highly resistant to the available drugs [30]. During the lifetime of an individual, these dormant forms can reactivate themselves in conditions in which the immune system of the host is compromised. Therefore, addressing dormant *M.tb* is critical for the eradication of TB. For decades, researchers have worked on identifying the sites in which these dormant bacilli hide and the underlying mechanisms to design new therapeutic interventions to target these dormant bacilli. In this regard, MSCs have recently been identified as a niche for dormant *M.tb*.

Although many studies have shown the involvement of MSCs during TB, the first link came from the study by Raghuvanshi et al. which showed that *M.tb* recruited MSCs at different sites during infection, such as the periphery of the granulomas and spleen in an infected mouse model. The presence of MSCs in the granuloma was shown to influence the microenvironment of the granuloma by regulating various aspects of T-cell functions, thereby aiding the survival of *M.tb*. In the granuloma, MSCs were shown to secrete multiple immunosuppressive molecules, such as nitric oxide (NO), which inhibited T-cell responses. Apart from directly inhibiting T-cell responses, MSCs were also shown to induce the development of T regulatory cells. NO is also an effective antibacterial molecule that was shown to restrict bacterial growth but could not eradicate latent bacilli. This dual function of MSCs was critical, as *M.tb* is present in the core of the granuloma, and MSCs uniquely position themselves, creating an equivalence zone to facilitate the survival of latent bacteria in the granuloma. Additionally, the presence of MSCs that were positive for the marker CD29 was observed in lymph node biopsies from patients with tubercular lymphadenitis [31]. This study was critical in shedding light on the involvement of MSCs in TB.

Later, Das et al. showed that *M.tb* infected CD271^+^ BM-MSCs in vitro and induced persistence in the nonreplicating dormant state. The researchers also showed that dormant *M.tb* retained its viability, and the undifferentiated state of MSCs was required for the viability and retention of *M.tb*. The researchers further showed the presence of *M.tb* in the bone marrow of a mouse model of TB. To examine the reinfection capabilities of dormant *M.tb*, the researchers used a specific mouse model in which they showed that the dormant *M.tb* in the lung and bone marrow MSCs could grow again when cultured and had the capability to infect the mouse. The presence of dormant *M.tb* in the bone marrow was also shown in TB patients who underwent anti-TB therapy. These findings suggested that the persistent *M.tb* in MSCs could be a possible source of reinfection [32]. Because viable *M.tb* residing in MSCs could be recovered from individuals who had completed anti-TB therapy, MSCs provide a niche that is resistant to antimycobacterial drugs. Additionally, it was shown that after infection in a mouse model, *M.tb* not only establishes infection in the lungs and spleen but also moves to the bone marrow between 7 and 14 days after infection and resides in BM-MSCs. Furthermore, the growth kinetics were shown to be different between the lungs and the bone marrow. The bacteria initially showed rapid growth in the lungs, followed by a significant decline as soon as the host immune responses were active. In contrast, the *M.tb* residing in the bone marrow did not show rapid growth but maintained a low number throughout. The dormant bacilli residing in the BM-MSCs were resistant to anti-mycobacterial drugs such as isoniazid and rifampicin and were a source for reinfection [33]. Another study showed that the *M.tb* present in MSCs was resistant to anti-TB drugs, and this phenomenon was attributed to the expression of a large number of ABC drug efflux pumps, such as ABCG1 and ABCG2 [34].

Furthermore, a study showed the possible localization of these *M.tb*-containing MSCs in the bone marrow. The results revealed that these MSCs resided in the hypoxic niche of the bone marrow. GFP-labeled H37Rv was present in BM-MSCs that were positive for pimonidazole after successful treatment with anti-TB drugs. Pimonidazole is a compound that forms stable adducts with cytosolic proteins in a hypoxic microenvironment. The cells containing these adducts could be detected by flow cytometry using antibodies against pimonidazole. Therefore, the in vivo administration of pimonidazole in the mouse model characterized the hypoxic niche of *M.tb*-infected MSCs. Furthermore, the researchers validated these findings in posttherapy TB patients. Since pimonidazole cannot be administered to posttherapy TB patients, a combination of the hypoxic markers HIF-1α and CD146 was used to identify the hypoxic niche of CD271^+^ BM-MSCs isolated from treated patients. RNA was isolated from BM-MSCs that showed the presence of *M.tb* DNA after treatment. Increased expression of HIF-1α and decreased expression of CD146 were observed, indicating the hypoxic niche of these BM-MSCs [35].

### Intracellular interactions of *M.tb* within MSCs

After entry into the host cell, *M.tb* can interact with different host proteins to reprogram the various metabolic and signaling pathways to evade the immune response or make the cellular environment favorable for survival. Various studies have shown the different pathways or molecules modulated by *M.tb* during infection in MSCs.

Fatima et al. showed the differential exploitation of MSCs relative to macrophages, which are the primary host, by *M.tb*. The scavenger receptors MARCO and SR-B1 have been reported to be involved in the phagocytosis of *M.tb* [36]. Upon entering MSCs, *M.tb* induces changes in the transcriptional signatures of various genes of its own and of the host. The expression of dormancy-related genes such as the DevS/devR regulon was induced, and *M.tb* was shown to remain in a dormant state, which was consistent with previous studies. However, in macrophages, replication-related genes of *M.tb* were highly expressed. A similar pattern was observed when macrophages and MSCs were isolated from *M.tb*-infected mice. Additionally, *M.tb* infection induced a state of quiescence in MSCs, which was evident by the increased expression of quiescence-related genes in MSCs, such as FOXO3A, NOTCH, and SOX9. On the other hand, genes involved in the proliferation of MSCs, such as SKP2 and CCNA1, were downregulated. The different pathways and the molecules of the host and *M.tb* that are involved in inducing these changes need to be further examined. The subcellular localization of *M.tb* within macrophages and MSCs was also different. *M.tb* was shown to exploit the host lipid synthesis pathway, creating a hideout in the lipid droplets in the cytosol of MSCs, whereas *M.tb* was mostly present in early endosomes after infection in macrophages. Induction of autophagy using rapamycin was effective in eliminating active and latent *M.tb* in vitro and in vivo in mouse models. These findings could be crucial for developing molecules targeting dormant *M.tb* [29].

**Fig. 3:**
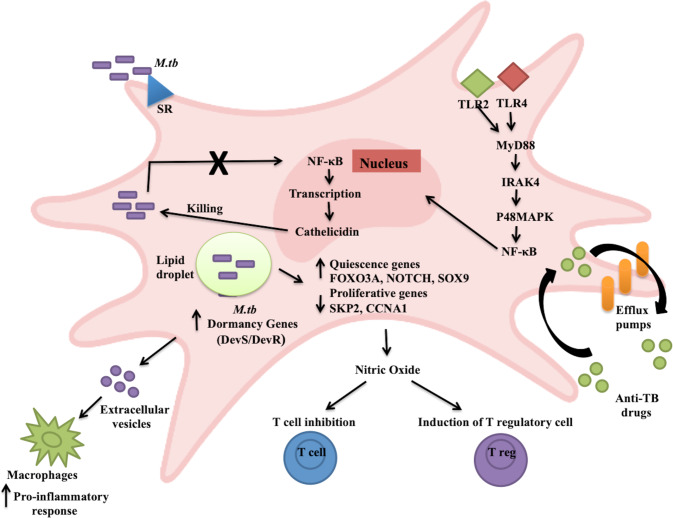
Various pathways induced in MSCs after *Mycobacterium tuberculosis* infection. *M.tb* uses the scavenger receptor expressed by MSCs to facilitate its entry. After entering MSCs, dormancy-related genes are expressed by *M.tb* and increased the expression of quiescence genes in MSCs while downregulating proliferative genes. Thus, *M.tb* is present in a dormant form inside lipid droplets within MSCs. *M.tb* also suppresses TLR2/TLR4-mediated activation of MyD88/IRAK4/P48MAPK/NF-κB and the subsequent transcription of cathelicidins, thereby avoiding killing mechanisms. MSCs express efflux pumps that protect *M.tb* from anti-TB drugs and secrete nitric oxide, which can inhibit T-cell responses. The extracellular vesicles secreted by *M.tb*-infected MSCs can induce a proinflammatory response in macrophages after being taken up

Another study illustrated the mechanism by which *M.tb* evades the antibacterial responses in MSCs and aids their survival. Cathelicidin, an antimicrobial peptide (AMP), is a crucial antibacterial factor in MSCs. Naik et al. showed that MSCs could restrict the growth of avirulent Mycobacteria such as *M. bovis* BCG and *M. smegmatis* but not that of virulent *M.tb*. The survival of *M.tb* was due to the downregulation of cathelicidin. Additionally, the researchers also showed reduced expression of proinflammatory cytokines such as TNFα and IL-1β and enhanced expression of anti-inflammatory cytokines such as IL-10 after infection of MSCs with *M.tb*. They showed that MSCs readily killed avirulent BCG by activating the TLR2/TLR4-mediated Myd88-IRAK4 activation of the p38 MAPK pathway, resulting in the activation and nuclear translocation of NF-κB and activating proinflammatory cytokines and cathelicidins. In the case of *M.tb*, cathelicidin and proinflammatory cytokine expression were downregulated, suggesting a mechanisms by which virulent *Mycobacteria* can escape from the killing mechanisms of MSCs, resulting in persistence [37] (Fig. 3). However, the components of the pathways targeted by *M.tb* and the use of cathelicidin to develop host-directed therapies against TB need further exploration.

### Role of MSC-derived exosomes in TB

Although a few mechanisms by which *M.tb* can persist in MSCs have been discovered, the field still requires intensive investigation. It is necessary to understand the pathways and crucial host factors involved in establishing *M.tb* dormancy to develop newer strategies for diagnosis and host-directed therapies. To develop better diagnostics and treatments for latent TB, the cargo of exosomes secreted by *M.tb*-infected MSCs should be investigated. The exosome is a type of extracellular vesicle secreted by various eukaryotic cells. It is 30 to 150 nm in diameter, carries different biomolecules such as nucleic acids, proteins, carbohydrates, and lipids, and is involved in cell-to-cell communication [38–40]. Exosomes are important in regulating host immune responses during various infections [41]. Liu et al. demonstrated the proinflammatory effects of exosomes on macrophages. The researchers showed that exosomes secreted by *M.tb*-infected MSCs were readily taken up by macrophages and upregulated proinflammatory molecules such as tumor necrosis factor-α (TNF-α), RANTES, and iNOS compared with exosomes produced by uninfected MSCs. The maximum level of induction was observed in response to exosomes secreted after 72 h of infection. Further analysis revealed that the MAPK and NF-κB pathways induced proinflammatory responses, as observed by enhanced phosphorylation levels of p38 and IκB-α. When macrophages from TLR2-, TLR4-, or MyD88-knockout mice were incubated with the exosomes of *M.tb*-infected MSCs, there was no induction of the proinflammatory response, highlighting the crucial role of the TLR-MyD88 pathway in exosome-mediated induction. The in vivo effect of the *M.tb*-infected MSC-derived exosomes was also demonstrated by intranasal administration of these exosomes in C57BL/6 mice. IL12p40 and TNF-α levels were enhanced in the lungs of mice after exosome treatment, along with increased infiltration of neutrophils compared to those in the mice treated with PBS or the exosomes of uninfected MSCs [42]. Further studies are required to identify the different molecules in the exosomes secreted by *M.tb-*infected MSCs, which could further be used to understand the mechanisms involved in establishing dormancy or developing vaccines or diagnostic approaches.

### Therapeutic role of MSCs in TB

Although *M.tb* can infect MSCs and hide from the immune system, some studies have indicated the use of MSCs as an adjunct therapy for treating TB. One such study showed the safety and efficacy of autologous MSCs derived from bone marrow as an adjunct therapy for anti-TB drugs against multidrug- and extensively drug-resistant TB. A total of 30 patients with confirmed MDR or XDR TB were given MSCs (1 ×10^6^/kg) within four weeks of the initiation of treatment. A more robust immune response was observed after treatment with MSCs, and there were no severe side effects or complications, indicating the safety of MSC infusion. The secretion of cytokines from PBMCs was enhanced in response to MSC infusion, and the ability of T cells to respond when stimulated with IL-2 or IL-7 improved. This result was indicated by STAT5 phosphorylation. This improved ability to respond was associated with improved cytokine production. Thus, MSC infusion was safe and effective in these TB patients [43]. Another study showed the efficacy of bone marrow-derived MSCs as an adjunct treatment for bladder TB. They showed reduced bladder deformation and inflammation and improved regeneration of the wall of the bladder after MSC infusion in a rabbit model [44]. Although these studies assessed the safety and effectiveness of MSCs in TB, further trials with a large number of patients are necessary to determine the use of MSCs in TB treatment.

## MSCs and other bacterial infections

In addition to TB, there are numerous bacterial infections that lead to a heightened risk of mortality and morbidity worldwide. Extensive research has been conducted describing the role of MSCs in other bacterial diseases. MSCs protect against acute pulmonary infection caused by *Streptococcus pneumoniae*. Asami et al. showed the anti-inflammatory role of MSCs against *Streptococcus pneumoniae*. In vitro, bone marrow-derived macrophages (BMDMs) stimulated with TLR2 and TLR9 ligands were cultured in the presence of MSC-conditioned media, which downregulated proinflammatory cytokines such as IL-6 and TNF-α and induce the overexpression of the anti-inflammatory cytokine IL-10. The study also showed the suppression of neutrophil chemoattractants such as CXCL1 and CXCL2. Similarly, intravenous administration of MSCs in a mouse infected with *Streptococcus pneumoniae* resulted in a reduced bacterial burden, increased expression of anti-inflammatory cytokines, and reduced neutrophil infiltration in the lungs [45].

Other studies have reported the anti-inflammatory benefits of MSCs in bacterial infections. A study showed the beneficial effects of MSCs on endotoxin-induced acute lung injury. The researchers showed that introducing MSCs into the lungs of mice that were administered *E. coli* endotoxin led to better survival than in control mice. This outcome was attributed to a decrease in proinflammatory mediators such as TNF-α and MIP-2 and increased levels of IL-10 [46]. Similar results of improved survival in mice administered MSCs with *E. coli*-mediated pneumonia have also been demonstrated. The improved clearance of bacteria after MSC administration was due to increased expression of the antibacterial protein lipocalin-2 [47]. MSCs have also been effective against an important bacterium that is primarily responsible for causing nosocomial infections: *Klebsiella pneumoniae*. MSCs were effective treating in acute lung injury by reducing the expression of TNF-α and expanding IL-17- and IFN-γ-producing T subsets [48].

Along with the immunomodulatory nature of MSCs, many reports have indicated the direct antibacterial nature of these cells [49]. The antimicrobial effects of MSCs through antimicrobial peptides have been supported by several in vitro and in vivo studies. A study on equine MSCs and bacteria commonly found in wound infections, such as *E. coli* and *S. aureus*, examined this property of MSCs. MSCs and their conditioned media could inhibit the growth of these bacteria and reduce membrane integrity. These antibacterial effects were due to the antimicrobial peptides cystatin C, elafin, lipocalin2, and cathelicidin in the MSC secretome and other factors [50, 51].

MSCs have also been effective against bacterial biofilms by inhibiting biofilm formation or disrupting the biofilm structure. MSCs represent a novel therapeutic model, since this biofilm is not easily located and is highly resistant to available antibiotics. Studies have shown that persistent biofilms of *Staphylococcus aureus* can be inhibited by administered activated MSCs as an adjunct with antibiotics or by augmenting pathogen clearance through cathelicidin LL-37 production by MSCs [52]. LL-37 is an amphipathic antimicrobial peptide that promotes bacterial lysis by targeting lipopolysaccharide and the endotoxin released by bacteria. Another study showed that the secretome of equine MSCs could inhibit and destabilize biofilms of methicillin-resistant *S. aureus* (MRSA) and other wound-related bacteria. The researchers demonstrated that the MSC secretome in the culture supernatant could inhibit and disrupt the mature biofilm. This effect was achieved mainly by protein degradation, since the secretome consists of various cysteine proteases associated with cathepsins. Pretreatment of these biofilms with the secretome also increased the efficiency of antibiotics, which were otherwise ineffective [53]. Another study by Chow et al. showed similar antibiofilm activity of MSCs. The direct antibacterial activity of MSCs was induced by the growth inhibition of *E. coli* and *S. aureus* cultured in the presence of MSC-conditioned media (CM). MSC-CM also enhanced the effectiveness of conventional antibiotics. This treatment increased the phagocytic activity of neutrophils[54]. There have been a few piecemeal studies suggesting the regulation of the immunosuppressive behavior of MSCs by chemotactic activation of TLR. Additionally, β-defensins secreted by MSCs aid in the clearance of gram-negative bacteria [55]. Microarray analysis of MSCs was performed to identify the antibacterial effects of β-defensin-2 and TLR-4 against *E. coli*-induced acute lung injury in mice [56] (Table 1).

**Table 1:** Immunomodulatory and antibacterial effects of mesenchymal stem cells against various bacterial infections

Bacteria	Type of MSCs or component used	Effects	References
*Streptococcus pneumoniae*	Mouse bone-marrow MSCs and MSC-conditioned media	Downregulation of pro-inflammatory cytokines IL-6, TNF-α, Upregulation of anti-inflammatory cytokine IL-10, Reduced Neutrophil infiltration, Reduced expression of Neutrophil chemoattractants CXCL1 and CXCL2.	[45]
*Klebsiella pneumoniae*	Mouse bone marrow MSCs.	Reduced expression of TNF-α, decreases in IL-17 and IFN-γ^+^ T-cell subsets.	[48]
*E.coli*	Mouse bone marrow MSCs Equine MSCs and MSC-conditioned media	Decrease in the proinflammatory mediators TNF-α and MIP2, increased expression of IL-10, increased expression of lipocalin2 Antibacterial effects due to cystatin C, lipocalin2, elafin, Cathelicidin	[46, 47] [50, 51]
*Staphylococcus aureus*	Equine MSCs and MSC-conditioned media	Antibacterial effects due to cystatin C, lipocalin2, elafin, Cathelicidin Degradation of biofilms due to cathepsins Increased phagocytic activity of neutrophils.	[50, 51] [53, 54]

## Role of mesenchymal stem cells in viral diseases

The immunoregulatory effect of MSCs has been beneficial in many viral diseases [57]. For instance, the infusion of umbilical cord-derived MSCs into patients with hepatitis B virus-related acute-on-chronic liver failure (ACLF), plasma exchange and the antiviral drug entecavir improves the survival rate and decreases the severity of infection [58]. Total serum bilirubin, albumin, alanine aminotransferase, aspartate aminotransferase levels and the model for end-stage liver disease scores were also improved in MSC-treated patients. A similar study using bone marrow-derived MSCs in which peripheral infusion with 1-10 ×10^5^ cells/kg was performed showed similarly improved patient survival [59].

MSC-derived extracellular vesicles (EVs) also exhibited anti-influenza and anti-inflammatory properties in vitro and in a preclinical pig model of influenza. Incubating MSC-derived EVs with lung epithelial cells inhibited viral replication and virus-induced apoptosis. Inhibition of the hemagglutination activity of avian, swine, and influenza viruses was also observed. These results were replicated in a pig model of the influenza in which intratracheal administration of MSC-derived EVs reduced viral replication, the secretion of proinflammatory cytokines, and viral shedding [60].

Zhang et al. evaluated the effectiveness of umbilical cord MSCs in restoring the immune deficiency of HIV-infected individuals who were immune nonresponders (INRs). After highly active antiretroviral therapy (HAART), some HIV-infected patients fail to restore CD4^+^ T cells and are highly susceptible to opportunistic infections even after complete viral suppression and are termed immune nonresponders. Infusing MSCs in these patients increased naïve and central memory CD4^+^ T cells and HIV-specific IFN-γ and IL-2 production. Thus, MSCs were safe and could be used as a restoration therapy for immune deficiency in HAART-treated HIV patients [61]. Qian et al. illustrated the use of umbilical MSC-derived exosomes to treat hepatitis-C (HCV) infection. MSC-derived exosomes inhibited viral replication in vitro without any cell toxicity. These effects of MSC-derived exosomes were due to the presence of miRNAs such as let-7f, miR-145, miR-199a, and miR-221. These factors also possessed binding sites for HCV RNA. These MSC-derived exosomes synergistically acted when combined with an FDA-approved drug (VX-950) to treat HCV infection. When Huh7 cells, a liver cancer cell line infected with HCV, ere cultured in the presence of MSC-derived exosomes, IFN-α and VX-950, the inhibition of viral infection was more potent, supporting the use of MSC-derived exosomes as adjuvant therapy with existing HCV drugs [62].

Although MSCs have been beneficial in virus-associated therapies, many reports have suggested that certain viruses can infect MSCs. Infection can also modulate anti-inflammatory, antimicrobial, and other properties of MSCs. This is a crucial factor when using MSCs as a therapeutic agent. Few reports have suggested the role of MSCs as a reservoir of HIV infection [57, 63]. Meisel et al. showed that in response to infection with cytomegalovirus, the inhibitory effect of MSCs on T-cell proliferation was partially reversed. Furthermore, it was observed that in response to CMV infection, the synthesis of IFN-γ-induced indolamine-2,3-dioxygenase (IDO), which is responsible for the inhibitory effects of MSCs on T-cell proliferation, was inhibited. Additionally, the researchers demonstrated that the antimicrobial effects of MSCs were inhibited by CMV infection [64]. MSCs can also be infected with hepatitis B virus [65] (Table 2).

**Table 2:** Immunomodulatory effects of mesenchymal stem cells against various viral infections

Viruses	Type of MSCs or component used	Effects	References
Hepatitis B virus	Umbilical cord MSCs and Bone marrow MSCs	Increased bilirubin, albumin, alanine aminotransferase, aspartate aminotransferase and a model for end-stage liver disease scores.	[58, 59]
Hepatitis C virus	Umbilical cord MSC-derived exosomes	Inhibition of viral replication.	[62]
Influenza Virus	Swine bone marrow MSCs extracellular vesicles	Inhibition of viral replication and hemagglutination activity, decreased levels of proinflammatory cytokines.	[60]
Human Immunodeficiency virus (HIV)	Umbilical cord MSCs	Increased naïve and central memory CD4^+^ T cells, high levels of HIV-specific IFN-γ, and IL-2 production.	[61]

## MSCs and malaria

Malaria is a protozoan disease caused by several species of the genus *Plasmodium*. It is the most common disease prevalent in developing nations such as Africa and many regions of Asia [66]. Human malaria is caused by five species of the *Plasmodium* genus: *P. falciparum*, *P. malariae*, *P. vivax*, *P. ovale*, and *P. knowlesi*. Transmission between humans occurs through the female Anopheles mosquito. The life cycle of Plasmodium is highly complex and is divided into a sexual phase that occurs in the mosquito and an asexual phase that occurs in the vertebrate host [67]. Despite the presence of many therapeutic strategies, malaria is still prevalent \ due to the development of drug-resistant species. Therefore, new interventions and strategies are urgently required to eradicate malaria. Many studies have examined the role of MSCs during malaria pathogenesis, which could help design new therapies.

Thakur et al. showed that infection with *Plasmodium berghei* induces splenomegaly in mice, and large numbers of accumulating cells are MSCs. When these MSCs from infected animals were adoptively transferred to naïve syngeneic mice followed by infection with the parasite, the mice acquired resistance and cleared the infection within 25 days. The levels of hemozoin in these animals were also low, which corresponded to a low level of parasitemia. The MSCs that confer protection against malaria are proinflammatory, as evidenced by high serum levels of proinflammatory cytokines such as IL-6, IL-12, IL-1β, and TNF-α. MSCs also inhibit the activation of regulatory T cells and the accumulation of NKT cells in the spleen [68].

Another study in which bone marrow MSCs were intravenously introduced in a *P. berghei*-infected mouse model of experimental cerebral malaria exhibited similar results. MSC infusion enhanced mouse survival and resulted in low parasitemia compared to those in control mice. Although treatment did not improve brain function in the MSC-infused mice, the histological architecture in other affected organs, such as the spleen, liver, lung, and kidney, was enhanced. In the lungs, functional capacity also improved. However, the number of astrocytes and oligodendrocytes in the brain as increased, indicating an increase in tissue repair in treated mice [69].

Anemia is a critical outcome of malaria infection that results from the destruction of parasitized erythrocytes during the schizont stage when the cells rupture. However, the destruction of nonparasitized erythrocytes is also a major contributing factor to the severity of malarial anemia [70]. The inability to replenish these erythrocytes due to dysregulated and ineffective erythropoiesis worsens the situation [71]. A critical study by Thakur et al. elucidated the role of MSCs in erythropoiesis and showed that MSC infusion in mice promoted the proliferation and differentiation of bone marrow cells into erythroid lineages. After the mice were infused with MSCs, an increase in the CD34^+^ population and CFU-E cells (later-stage erythroid progenitor cells) was observed. Additionally, the levels of the crucial erythroid-specific transcription factors GATA-1 and GATA-2 were upregulated after MSC infusion, resulting in increased erythropoiesis. The CD4^+^ T and CD8^+^ T-cell populations increased, and the expression of PD-1 was low [72].

## Priming MSCs for immune modulation

Various studies have shown that MSCs modulate host responses in different conditions. Recently, the idea of preconditioning MSCs to achieve target-specific properties has been under investigation. Preconditioning MSCs involves activating or priming the cells with extraneous substances, exposing them to loaded extracellular vesicles or culturing them in heterogeneous conditions. Studies have indicated that preconditioning MSCs with various stimuli, such as hypoxia, nutrient starvation, oxidative stress, and heat shock and supplementation with potent drugs and inhibitors enhances their therapeutic functions. MSC preconditioning modulates cytokine and chemokine secretion and increases their movement to the target site or secretion antimicrobial peptides, which plays a crucial role in host defense against bacterial and viral infections. MSC preconditioning with various factors is critical in determining their immunomodulatory effects, which are critical in mycobacterial infection. A study showed the proinflammatory nature of mouse bone marrow-derived MSCs that were pretreated with poly(A:U) and then introduced intravenously into BCG-infected mice. The results showed that when poly(A:U)-treated MSCs were administered, the levels of proinflammatory cytokines such as TNF-α and IL-6 increased significantly, splenomegaly was reduced, and a decrease in bacterial growth was observed. The effects were contrasting in mice into which naïve MSCs were introduced, and these cells were anti-inflammatory and favorable for bacterial growth [73].

### Preconditioning with hypoxia

In vivo, MSCs reside in hypoxic niches; thus, their response to various drugs and stimulants depends on oxygen levels. In vitro, MSCs are cultured in 20-21% oxygen (normoxia), while in vivo, the oxygen level is approximately 1-5% (hypoxia). The striking difference in oxygen concentrations modulates their responses. HIF-1α, a transcription factor associated with hypoxia and hypoxic conditions, was shown to be upregulated. HIF-1α acts as a low oxygen sensor and is involved in autocrine and paracrine signaling, enhances the production of vascular endothelial growth factor (VEGF) and fibroblast growth factor-2 (FGF-2), and leads to increased angiogenesis and proliferation [74]. The induction of HIF-1α/2α protects against hypoxic stress and downregulates p53 levels [75]. In umbilical cord MSCs, Bicaudal D homolog 1 (BICD1) regulates dynein-mediated HIF-1α nuclear translocation. BICD1 targets GSK3β, which activates glycolysis, enhancing the survival and proliferation of MSCs under hypoxic conditions [76]. Hypoxia also activates Caspase-3, which activates proinflammatory signaling. A recent study on MSC spheroid models showed that hypoxia preconditioning induced HIF-1α expression and enhanced the therapeutic effects on bone formation and repair [77]. Another study showed that hypoxic MSCs activated miR-211, which positively regulates the transcription factor STAT3, leading to increased migration under hypoxic conditions [78].

Interestingly, another study suggested that hypoxic preconditioning enhanced the retention of transplanted MSCs in vivo, thus maximizing the target effect in injected mice. These MSCs limit glucose absorption in serum deprivation conditions and, therefore, consume glucose through glycolysis and secrete relatively less lactate into the supernatant than control MSCs [79]. Hypoxic-preconditioned MSCs inhibit senescence and support their mesenchymal stem cell properties by downregulating E2A-p21, which plays a crucial role in epithelial-to-mesenchymal transition in vitro. P21 activation induces cell senescence and apoptosis. HIF-1α is associated with TWIST, a transcription factor that regulates p21, which prevents senescence and apoptosis and maintains stem cell properties in MSCs [80]. These findings suggest that hypoxic-preconditioned MSCs play a promising role in various diseases. However, further investigation is needed to determine the effectiveness of these MSCs in diseases and immune modulation.

### Priming with immunomodulatory factors

MSCs possess short-term memory of the environmental responses in their environment. Thus, they remember the stimulus from their previous environment. This property of MSCs has been used to exert targeted therapeutic effects. When MSCs are primed with cytokines, they show increased immune modulation. A study showed that TNF-α-exposed MSCs secreted exosomes that enhanced regeneration potential compared to control MSCs [81, 82]. A study of a neonatal rat model of sepsis preconditioned hUC-MSCs that were treated with IFN-γ and later infected with *E. coli*. The preconditioned UC-MSCs enhanced bacterial clearance and increased the survival of rats compared to saline preconditioned UC-MSC-treated rats [83]. The critical role of MSCs has been studied not only in rats and mice but also in equines. Priming equine MSCs with IFN-γ and TNF-α induced PGE_2_, indoleamine 2,3-dioxygenase, iNOS, and IL-6 expression. This activation induced an anti-inflammatory response and inhibited lymphocyte proliferation [84]. Human periodontal ligament stem cells (PDLSCs), which are similar to MSCs, were preconditioned with lipopolysaccharide (LPS), and the conditioned media induced M1 macrophage polarization [85]. The priming of MSCs shows encouraging results in MSC therapies. However, further investigation is required to produce long-lasting therapeutic effects of primed MSCs.

## Interactions of MSCs with the host immune system

The immunomodulatory effects of MSCs and their involvement in various infectious diseases result from their interactions with different immune cells in the body. In TB, the granuloma is a well-defined structure composed of macrophages, neutrophils, MSCs, B-lymphocytes, and T-lymphocytes [86]. There is little knowledge of the interactions of MSCs with different immune cells within the granuloma, which is a major obstacle in understanding the mechanism of the establishment of latency. Raghuvanshi et al. showed the presence of MSCs at the periphery of the granuloma and their interaction with T cells, and it is worth examining whether MSCs also interact with other immune cells, such as B cells, macrophages, dendritic cells, and neutrophils, to maintain an immune-privileged environment for *M.tb*. Thus, in this section, we have highlighted the present knowledge and evidence on the interplay between MSCs and different immune cells that can be used to determine incomplete areas for a better understanding of the immune-privileged niche for *M.tb* within the host.

As previously mentioned, MSCs have reduced expression of MHC-I and lack MHC-II and costimulatory signals such as CD80, CD86, and CD40. These cells can modulate the innate and adaptive immune systems. This immunomodulation ability is mediated by the interactions of MSCs with different immune cells, including T cells, B cells, NK cells, dendritic cells, macrophages, and neutrophils, due to the anchorage of numerous adhesion molecules, such as lymphocyte function-associated antigen-3 (LFA-3), intercellular cell adhesion molecule- 1 (ICAM-1), and vascular cell adhesion molecule (VCAM)-1, and the production of soluble factors in various microenvironments [87, 88].

In addition, immune modulation by MSCs involves numerous factors, such as cytokines and growth factors, to modify and stabilize the immune profile. This is further achieved by the activity of the soluble secretome, including indoleamine 2,3-dioxygenase (IDO), nitric oxide (NO), or prostaglandin E_2_ (PGE-2) [87]. In addition to these factors, adhesion molecules and major histocompatibility complex (MHC) molecules are required to mediate immune suppression [88]. Additionally, the MSC-induced complement system modulates the immune response by activating myeloid effector cells and initiating a cascade of reactions [89]. The crosstalk between MSCs and various immune cells depends on the environment and pathological or physiological conditions in which they are present, such as repair, regeneration, transplantation, or infection. MSCs secrete proangiogenic components during tissue rejuvenation, stimulating neovascularization through juxtracrine and paracrine signaling [90]. In vivo and in vitro experiments have demonstrated the suppressive behavior of immune cells in response to MSCs. However, MSCs influence activation and inhibition pathways depending on the exposed antigen [91]. Interestingly, a study was performed that showed the immune-enhancing effect of MSCs through the inadequate production of nitric oxide (NO).

### MSC interactions with T cells

T cells are the central regulators of the adaptive immune system with the potential to encounter diverse foreign antigens and differentiate to maintain the immunological response. Naïve T cells are activated in response to acknowledging their specific antigen presented by MHC molecules as peptides, and these cells proliferate to maintain long-term immunity and self-tolerance [92].

A study showed that gingiva-derived MSCs could inhibit the proliferation of CD4^+^ T cells through CD39/CD73-mediated production of adenosine and the IDO pathway [93]. There is evidence that indicates an immunosuppressive response mediated by T cells when in contact with MSCs. This response is generally shown to be induced by the secretion of specific cytokines such as IFN-γ and TNF-α [94]. Studies have shown that IFN-γ secreted by proinflammatory T cells facilitates the inhibition of RunX2 (Runt-related transcription factor 2), thereby upregulating TNF-α in extrinsically administered MSCs. This results in impaired bone regeneration activity by preventing apoptosis in mice [95].

In 2013, Najar et al. elucidated the immunomodulatory effects of tissue-derived MSCs and demonstrated that among all the types of MSCs, adipose tissue-derived MSCs (AT-MSCs) modulated T-cell activation markers, including CD69, CD45, CD26, and CD23. These MSCs induced anti-proliferative and anti-inflammatory effects by downregulating IFN-γ levels. AT-MSCs have been shown to significantly downregulate the expression of CD26 and CD45 and maintain sustained CD69 expression. AT-MSCs promote lymphocyte migration, which slows the interaction between T cells and MSCs, resulting in impaired lymphocyte proliferation. AT-MSCs increase IL-8 and CCl-5 secretion, enhancing the recruitment of T cells [96].

Another study has contributed to understanding the roles of other tissue-derived MSCs in immune modulation. Monocytes play a crucial role in mediating the suppressive effects of MSCs on T-cell proliferation. Umbilical cord-derived MSCs (UC-MSCs) induce rapid alterations in monocyte surface markers via PGE2, and treatment with indomethacin inhibited the suppression of T cell proliferation by UC-MSCs. It was also found that monocytes isolated from cocultures of UC-MSCs had significantly downregulated accessory cell and allostimulatory functions in T-cell proliferation assays [97]. Groh et al. carried out a similar study, and they examined how bone marrow-derived MSCs inhibited alloreactive T-cells. They observed that monocytes activated MSC to secrete inhibitory molecules such as IL-1β and TGF-β1 but not IL-10 to inhibit alloreactive T-cells. Other tissue-derived MSCs downregulate T-cell activation markers such as CD25, CD38, and CD69 [98]. Indoleamine-2, 3-dioxygenase (IDO) is an intermediate that mediates the immunomodulatory functions of MSCs in humans. MSCs induce IDO expression, which catabolizes tryptophan, leading to tryptophan starvation and thereby inhibiting T-cell proliferation [99]. Another aspect of MSC-mediated immunosuppression is correlated with Tregs during kidney transplants in patients. Tregs did not alter the functions of adipose tissue-derived MSCs; instead, they supported these MSCs by activating them and increasing their efficiency. This study suggested that MSCs induce IL-10 production in effector cells and Tregs by expressing IDO, thus exerting suppressive effects [94]. Although MSCs have been shown to exhibit immunosuppressive behavior, human MSCs have been shown to promote the survival of unstimulated T cells in their resting stage. However, MSC promotes apoptosis in activated T-cells by mitogenic, allogenic, or antigenic stimuli but decreases Th1 differentiation. This study showed the role of mesenchymal stromal cell-derived extracellular vesicles (MSC-derived EVs) in regulating T cell proliferation, and MSC-derived EVs induced the upregulation of Foxp3, thereby increasing the number of IFN-γ^+^/Foxp3^+^T cells [95]. Autophagy has also been shown to play a crucial role in the immunosuppressive functions of MSCs. Inhibiting autophagy with 3-methyladenine (3-MA) decreases the migration of T-cells, activating the autophagy pathway with rapamycin promotes CD4^+^ T cell migration, and CXCL8 and TGF-β1 secretion is enhanced by the activation of autophagy in MSCs. This is accompanied by an increase in Tregs and a decrease in Th1 cells [96]. Najar et al. demonstrated the role of the CD200/CD200R axis in MSC/T-cell cocultures in conferring immunosuppressive effects. They observed that among all the MSCs, Wharton’ Jelly (WJ)-derived MSCs abundantly express CD200 and may be a selective marker for these cells. The high abundance of these markers on MSCs may be due to the proinflammatory environment and IFN-γ. However, the functions of these surface markers on MSCs are yet known [100]. There are other receptors and ligands present on MSCs that regulate lymphocyte differentiation, migration, and proliferation. One such factor is the erythropoietin-producing hepatocellular (Eph) receptor tyrosine kinase family and their specific ephrin ligands. It was demonstrated that ex vivo MSCs expressed EphB2 and ephrin-B2, while T cells expressed their corresponding ligands: ephrinB1 and EphB4, respectively. In response to stimulation of these receptors, immunosuppressive factors were upregulated in MSCs, including IDO, TGF-β1, and iNOS. These factors inhibited T cell proliferation partly via activation of the Src, PI3 Kinase, Ab1, and JNK kinase pathways [101]. It is important to note that MSCs suppress T-cell proliferation and differentiation via multiple pathways, and each response is specific to the type of MSC targeting T cells.

### MSCs and B cells

B cells are crucial factors in adaptive immunity that develop in the bone marrow stroma. These cells undergo maturation in the bone marrow and play an essential role in antibody production, antigen presentation, and complement activation. It has been reported that MSCs modulate T cells, but their effects on B cells are unclear. MSCs can regulate the activation, differentiation, and function of mature B cells and impact regulatory B cells (Bregs) [102]. Limited efforts have been made to understand the role of MSCs in regulating B cells. A study in 2009 showed that MSCs suppressed B cells, specifically plasma cells, by secreting certain humoral factors and did not require direct cell-to-cell contact. It was found that Blimp-1 was reduced in B cells that were cocultured with MSCs, but the factors involved in this immune suppression were not clear [103]. Several studies have shown that hMSCs restrict B-cell proliferation by impairing the production of immunoglobulins and chemokines without restricting cytokine production. Multiple studies have shown the vital role of MSCs in modulating the functions of regulatory B cells [104, 105]. One study showed that MSCs could enhance the proliferation and functions of CD5^+^ regulatory B cells, such as the secretion of anti-inflammatory IL-10. IDO was partially involved in these effects of MSCs [93]. Another study demonstrated that MSCs could increase the number of CD5^+^ regulatory cells, which could produce IL-10 and inhibit T-cell responses [106]. MSC-derived EVs have been shown to exert immunomodulatory effects on B cells. It was found that MSCs regulated the induction of CD24^hi^CD38^hi^ IL-10-producing Breg phenotypes and naïve B cells but not their proliferation. MSC-derived EVs, on the other hand, do not promote naïve B cells and reduce memory cells. However, similar to MSCs, MSC-derived EVs induce CD24hiCD38hi B cells but not Bregs, since they do not produce IL-10 [107]. Another study demonstrated the effects of MSC-derived exosomes on the B-cell function by regulating the transcriptional landscape of B cells [108].

### MSCs and Natural Killer (NK) cells

Natural Killer (NK) cells are large bone marrow-derived granulocytes that are critical in mediating defense against tumors and microbial infections [109]. The presence of CD56 and the absence of CD3 are used to identify NK cells. It is well known that the regulation of these cells is controlled by the interplay between the activation and inhibition of neighboring cell receptors [110]. MSCs release soluble factors and cytokines to modify the functions of NK cells [111]. Similar to other immune cells such as T and B lymphocytes, MSCs prevent the proliferation of NK cells. This is primarily done by reducing IL-2/15-induced NK cell proliferation. In addition, the presence of MSCs leads to a significant reduction in cytotoxicity, IFN-γ production, and the levels of perforin and granzymes in NK cells. Moreover, MSCs downregulate the expression of surface receptors such as NKp30, NKp44, and natural-killer group 2 member D (NKG2D), which are typically responsible for NK cell activation and target cell killing. Additionally, immunosuppressive factors such as IDO, PGE2, and TGF-β participate in the inhibition of NK cells [112].

### MSCs and dendritic cells

Dendritic cells (DCs) are critical components of the immune system, and these cells activate various innate and adaptive immune cells [113, 114]. MSCs impact the maturation and activation of DCs and alter their functions, leading to the production of immature DCs. Numerous studies have shown that MSCs repress the differentiation of monocytes by suppressing the expression of CD1a, CD86, HLA-DR, and CD83 to inhibit the maturation of DCs [104]. One study showed that gingiva-derived MSCs inhibited the differentiation of DCs when cocultured with CD14^+^ monocytes and reduced the secretion of IL-12 in response to stimulation with LPS, and this effect was mediated by PGE2 [93].

### MSCs and macrophages

**Fig. 4:**
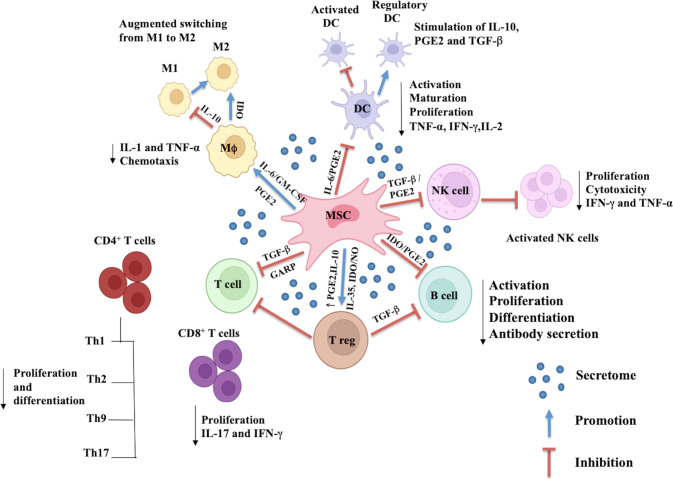
MSC-mediated immunomodulatory effects on specific immune cells. MSCs modulate innate and adaptive immune cells by positively and negatively regulating diverse juxtracrine and paracrine pathways via numerous cytokines and chemokines

Macrophages are the bridge between the innate and adaptive arms of the immune system. These are specialized cells capable of engulfing and eliminating foreign organisms and apoptotic cells through phagocytosis and efferocytosis, respectively. Understanding the interactions between MSCs and macrophages in TB could be crucial. Several studies have shown the crucial role of MSCs in regulating the polarization of macrophages, thereby influencing the microenvironment. One study showed that exposure of MSCs to alveolar macrophages stimulated the production of IL-10, TSG6, and PGE2 by MSCs, leading to macrophage reprogramming toward the anti-inflammatory M2 phenotype. In coculture conditions, MSCs enhanced macrophages expression of CD206, IL-10, and other anti-inflammatory mediators while significantly reducing proinflammatory cytokine expression. Furthermore, MSCs have been shown to increase macrophage phagocytic capacity [105]. Another study showed that the proinflammatory environment triggers MSCs to secrete anti-inflammatory factors, such as IDO, CCL18, and PGE2, thus mediating the polarization of macrophages from the M1 to the M2 phenotype. These factors interact with surface receptors on M1 macrophages and activate downstream signaling to initiate the polarization of M2 macrophages [104] (Fig. 4).

## Conclusion

Understanding the mechanisms by which *M.tb* can cause latency in the host to survive is critical for its eradication. In recent years, research has focused on identifying the various niches in which *M.tb* can hide in a dormant form and evade the various killing mechanisms of the immune system. As summarized in this review, mesenchymal stem cells have emerged as a favorable niche for dormant *M.tb*. MSCs are present in the granuloma, where they secrete various immunomodulatory molecules such as nitric oxide and create an environment in which they inhibit T-cell responses and restrict bacterial growth. After infecting MSCs, *M.tb* shows enhanced expression of dormancy-related genes and induces a state of quiescence in MSCs. MSC genes involved in proliferation are downregulated, and quiescence genes show enhanced expression. *M.tb* creates a reservoir in lipid droplets in the cytosol of MSCs. *M.tb* can also inhibit killing by antimicrobial peptides such as Cathelicidin. Apart from TB, MSCs also play a crucial role in other bacterial and viral diseases by interacting with various immune cells in the body. The immunomodulation by MSCs is mediated through various membrane-bound and soluble factors. Although multiple aspects of *M.tb*-MSC interactions have been discovered, the pathways and the crucial molecules involved in establishing dormancy and the factors that might induce the resuscitation of *M.tb* still need to be investigated. Identifying host and *M.tb* factors, such as miRNAs and proteins, that are critical for its survival in the MSCs could be significant in developing various therapeutic interventions and diagnostic tools for latent TB. Additionally, since *M.tb* residing in MSCs are tolerant to anti-TB drugs, new approaches such as phytochemicals or inhibitors that can target *M.tb* need to be explored. The two major roadblocks to eliminating TB are antibiotic resistance and the waning efficacy of currently approved vaccines. Thus, in this review, we aimed to highlight the features of the immune-privileged niche of *M.tb* that can be targeted to enhance the efficacy of current and newly developed antibiotics and vaccines. This is worth noting that *M.tb* develops smarter strategies to evade the host immune responses by surviving in unique host cells. Thus, exploring this immune-privileged niche may provide a better understanding of the immune evasion abilities of *M.tb* and aid in the design of adjunct therapeutic interventions to eliminate the bacteria.
